# Biliary Ascariasis in a Pediatric Patient in Lithuania: Case Report and Literature Review

**DOI:** 10.3390/medicina60060916

**Published:** 2024-05-30

**Authors:** Rūta Rokaitė, Mindaugas Dženkaitis, Melita Nedzinskaitė, Rūta Kučinskienė

**Affiliations:** 1 Department of Pediatrics, Medical Academy, Lithuanian University of Health Sciences, LT 44307 Kaunas, Lithuania; rutadrk@gmail.com; 2 School of Medicine, College of Health and Agricultural Sciences, University College Dublin, Belfield, D04 V1W8 Dublin, Ireland

**Keywords:** *Ascaris lumbricoides*, ascariasis, abdominal pain, bile ducts, child

## Abstract

Hepatobiliary ascariasis is caused by the entry of the nematode *A. lumbricoides* from the duodenum into the biliary duct. We report a case of an *Ascaris*-induced extrahepatic biliary tract obstruction in a pediatric patient admitted to the hospital due to a wide spectrum of symptoms of biliary disease, which included abdominal pain in the upper abdominal quadrants, vomiting, and jaundice. Imaging tests—including ultrasound, magnetic resonance cholangiopancreatography (MRCP), and endoscopic retrograde cholangiopancreatography (ERCP)—were performed to confirm the diagnosis of biliary ascariasis. The tests did, in fact, demonstrate signs of this disease. Nevertheless, during the ERCP, only the remains of *Ascaris* parasites in the form of tissue fragments were extracted. We also aim to discuss the prevalence of ascariasis in children, the causes of migration of *Ascaris* parasites into the bile ducts, together with its clinical manifestations, as well as the diagnostic and treatment methods of this disease.

## 1. Introduction

Ascariasis is a parasitic infection caused by Ascaris lumbricoides, a type of cylindrically-shaped roundworm. In accordance with the findings of the World Health Organization (WHO), it is estimated that a quarter of the global population is infected with soil-transmitted helminths (STH) [1], with ascariasis emerging as one of the most prevalent STH infections globally [2]. Even though ascariasis is found worldwide, the highest prevalence has been recorded in tropical and subtropical areas, i.e., in sub-Saharan Africa, China, South America, and Asia [1], where the warm and humid climate favors the spread of infection throughout the year. Ascariasis is common among both children and adults. The spread of this disease is significantly influenced by factors such as poor living standards, inadequate hygiene conditions, the absence of clean drinking water, consumption of contaminated food products, and improper household waste and sewage management [1,3].

In Lithuania, this parasitic disease accounts for about 12 percent of all officially registered helminth infections. In total, 200–300 cases of ascariasis are registered in Lithuania every year, with 90 percent of them being among children. Ascariasis infections are common in children who are 2–10 years of age [4]. The main reason for this is a lack of hygiene skills among this demographic (such as proper hand washing before meals or after spending time outside, etc.), which leads to a higher incidence of ascariasis.

Following ingestion via the oral route and completion of a full developmental cycle within the intestines, adult *Ascaris lumbricoides* parasites inhabit various parts of the gastrointestinal tract [5]. The disease is frequently diagnosed incidentally when the parasites are passed with stool or following stool analysis. Nevertheless, the disease might manifest nonspecific symptoms, including pain in the right upper abdominal quadrants, nausea, vomiting, and jaundice, [6] and cause severe complications, such as intestinal obstruction, biliary colic, cholecystitis, cholangitis, pancreatitis, hepatic abscesses, peritonitis, and Löffler’s syndrome [2,5]. 

Hepatobiliary ascariasis is a rare complication of ascariasis in nonendemic zones, especially in pediatric patients, while being quite common in endemic zones. Thus, physicians working with children should consider the possibility of *Ascaris*-induced lesions in the presence of signs of biliary obstruction, irrespective of the patient’s country of residence.

## 2. Case Report

An eight-year-old female patient residing in a small Lithuanian town was admitted to a tertiary-level university hospital due to severe acute pain in the upper abdomen and a bout of vomiting that lasted for one entire day. The symptoms had been recurring during the preceding 6 months. Following admission, laboratory tests showed elevated liver enzymes (ALT, AST, GGT), elevated α-Amylase, and hyperbilirubinemia, with direct bilirubin predominating. A dilated common bile duct (0.8–0.9 cm in size) and pancreatic duct (0.2 cm in size) were observed on abdominal ultrasound. An abdominal magnetic resonance imaging (MRI) test was performed and revealed no obvious cause of the bile duct obstruction. Following the implementation of symptomatic treatment, the patient’s general condition improved within several days. The resolution of abdominal pain and a normalization of laboratory test results were observed.

During the initial examination following a second episode of abdominal pain in the patient, only tenderness under the right side of the rib cage was noticed. No other pathological signs were found. Laboratory tests demonstrated elevated levels of transaminases (ALT, AST, GGT), elevated α-Amylase, total bilirubin, and immunoglobulin E (Table 1).

On the 1st day of the disease, a dilated common bile duct was observed in abdominal ultrasound (up to 0.9 cm in size), as well as dilated lobular ducts (0.5 cm in size). However, no dilation of intrahepatic ducts nor concrements within the gallbladder were observed. The symptoms disappeared within 4 days of treatment with spasmolytics, analgetics, and proton pump inhibitors, at which time no pathologies on the abdominal ultrasound were detected. During that time, viral autoimmune hepatitis, α1-antitrypsin deficiency, and Wilson’s disease were ruled out. Due to high concentrations of immunoglobulin E in the blood and a lack of a history of allergies, helminthiasis was suspected. As abdominal pain did not reoccur and all laboratory parameters had returned to their normal ranges, the patient was discharged from the hospital.

**Figure 1:**
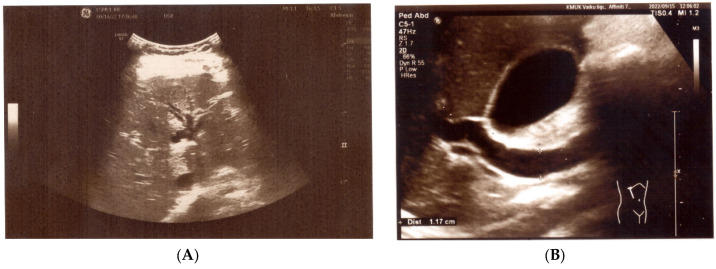
(**A**) US: dilated intrahepatic ducts. (**B**) US: dilated common bile duct.

The patient then underwent helminth testing at an outpatient clinic. *Ascaris lumbricoides* eggs and *A. lumbricoides* IgG antibodies were detected in the patient’s stool and blood, respectively. Subsequently, a third episode of the disease was observed. The patient experienced severe abdominal pain localized under the right half of the rib cage, accompanied by vomiting and jaundice within one-week post-discharge. Anti-helminthic treatment had not been initiated during this period. Laboratory tests demonstrated elevated total and direct bilirubin, transaminases (AST, ALT, GGT), α-Amylase, and eosinophils (Table 1). Abdominal ultrasound (US) was performed during this acute phase and some abnormalities were found: dilation of the biliary tree ducts and dilation of the common bile duct up to 0.8 cm and 1.17 cm in size, respectively (Figure 1A,B). Pancreas was of normal thickness and echogenicity, with the pancreatic duct being accentuated up to 0.2 cm along its entire length. The abdominal ultrasound showed no liver or gallbladder abnormalities.

**Figure 2:**
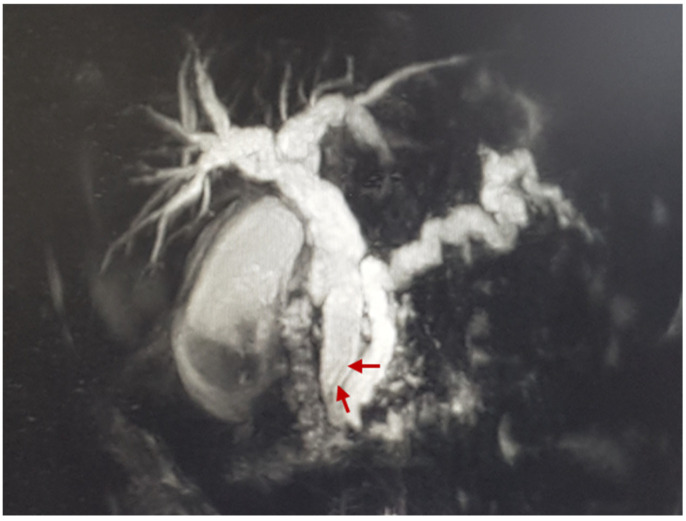
MRCP: Shadow of an *A. lumbricoides* parasite in CBD (red arrow).

Magnetic resonance cholangiopancreatography (MRCP) was performed. The test demonstrated the normal size and structure of the patient’s liver, with dilated hepatic and biliary ducts: the right hepatic duct (RHD) was ~0.9 cm in diameter, left hepatic duct (LHD) was ~1.0 cm, sectoral ducts were up to ~0.7 cm, and peripheral ducts were ~0.2 cm in diameter. The common hepatic duct (CHD) and common bile duct (CBD) were up to ~1.1 cm and ~1.2 cm in width, respectively, and filled with a cloudy fluid. CBD gradually narrowed up to a size of ~0.65 cm; at hiatus, CBD joined with the dilated common pancreatic duct, which demonstrated a visible filling defect measuring ~0.5 cm by 0.3 cm in the lumen. The gallbladder was enlarged and demonstrated many uneven, polycyclically contoured filling defects with a cloudy layer. The pancreas was homogenous, the pancreatic duct was curved along its entire length and enlarged to ~0.7 cm in size, and the secondary ducts were dilated (Figure 2).

**Figure 3:**
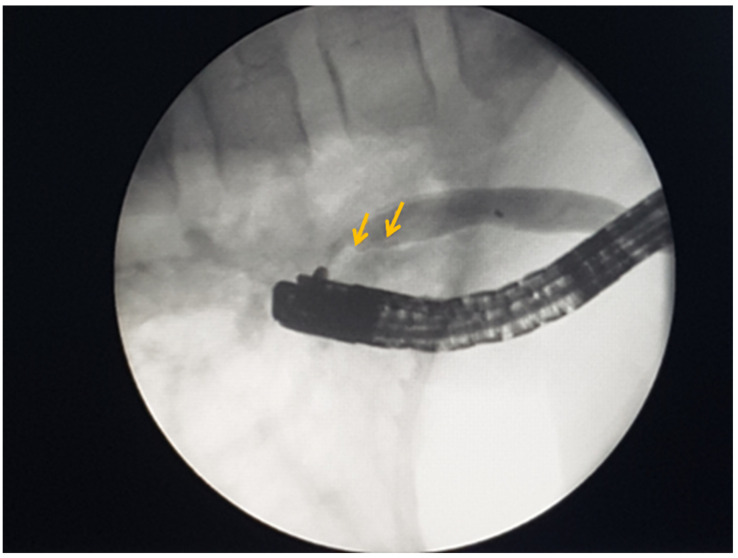
ERCP: CBD filling defects (yellow arrows).

Mebendazole was administered to the patient three days prior to her undergoing endoscopic retrograde cholangiopancreatography (ERCP). The ERCP demonstrated an elongated, endemic *p. Vateri* crease with a dilated sphincter and bloody bile. Biliary cannulation was unsuccessful. For this reason, a precut sphincterotomy was performed and deep CBD cannulation was repeated. Further, contrast material was injected, and a dilation with filling defects was detected (Figure 3). 

To preserve the integrity of the sphincter, a balloon dilatation of the CBD hiatus was performed with a 6 mm balloon, and CBD was revised. By using an extraction balloon, a few turbidities with black-colored inclusions were extracted, and the extracted material was determined to be the likely remains of an *A. lumbricoides* individual.

Mebendazole was swapped for a single dose of albendazole after 3 days due to the insufficient effect of mebendazole on *A. lumbricoides* in the gallbladder and the ducts, as mebendazole is metabolized in the liver to an inactive metabolite.

An abdominal ultrasound was performed a few days later. The gallbladder remained large in volume, without any filing defects visible on the ultrasound, biliary ducts were not dilated, CBD was 0.4 cm wide, and the common pancreatic duct was accentuated up to 0.2 cm in width.

The symptoms disappeared within a few days of the ERCP, with subsequent gradual normalization in the results of laboratory tests, leading to the patient’s discharge from the hospital. Scheduled regular follow-up examinations were conducted in an outpatient clinic closer to the patient’s place of residence. One year later, the patient remained asymptomatic, laboratory test results exhibited complete normalization, and ultrasound demonstrated a sustained enlargement of the gallbladder without any observable defects.

To our knowledge, this is the first case of pediatric ascariasis in Lithuania that developed biliary ascariasis as a complication.

## 3. Discussion

While ascariasis is presently acknowledged as a significant health concern in its endemic regions, it is imperative for physicians worldwide to be aware of this disease and its associated complications. This is crucial, as *Ascaris* have the capacity to infect individuals in non-endemic areas. Both adults and children suffer from ascariasis due to a lack of hygiene skills, though children develop *A. lumbricoides* infections more often than adults (60–90 percent of infected individuals are children) [4,7]. The literature suggests that the highest incidence of infection is among children 2–10 years of age and that the incidence declines in patients aged 15 years and above [2]. Research shows that the prevalence of ascariasis increases in the 2–3-year-old age group and becomes the highest at the age of 8–14 years [5]. In addition, children are more likely to have severe infection compared with adults (70 to 49 percent, respectively) in endemic regions [5]. In Lithuania, the highest percentage of ascariasis infections is among 2–10-year-olds. As expected, children in this age group exhibit deficiencies in their hygiene practices (proper hand washing before and after meals, picking up various dirty objects off the ground, etc.). Consequently, this results in a higher incidence of ascariasis within this age group [4]. Our patient also belonged to this age group of being at a high risk of an *A. lumbricoides* infection.

About 75 percent of the cases of ascariasis in Lithuania are diagnosed during prophylactic examinations, as patients usually experience mild to non-existent symptoms [4]. However, *A. lumbricoides* may sometimes cause various life-threatening complications. One of these complications is biliary ascariasis, which is more common among adults and occurs due to the migration of the helminth from the jejunum to the duodenum and through the ampulla of Vater to the biliary tree [8,9].

According to the literature, the incidence of adult biliary ascariasis is up to 36.7 percent [7], while reaching 10.0 to 19 percent among children in endemic regions [6,10,11]. Bile duct ascariasis is less common in pediatric patients due to them having narrower bile ducts.

*Ascaris lumbricoides* is normally found in the jejunum, although they have a natural tendency to migrate and seek out small openings all over the human body. Changes within the intestines, such as intestinal gas accumulation, increased pressure in the intestinal tract, and viral or bacterial co-infection of the intestinal tract, contribute to increased motility of the intestines. This further promotes the migration of parasites from their natural habitat in the jejunum to the duodenum and to the smallest parts of the intestinal tract (ducts) [7]. The host’s response to adult *A. lumbricoides* is the alteration of vasomotor reflexes and secretory responses, which affect gut tone and motility. The secretion of peptide cholecystokinin together with secretin is thought to decrease the tone of the sphincter of Oddi and facilitate parasite migration into the bile ducts. Roundworms secrete polypeptides which cause allergic reactions and spasms of the sphincter of Oddi. A chaotically stimulated sphincter of Oddi opens, and helminths, together with intestinal bacteria, migrate to the bile ducts [12]. Sometimes helminths ascend the bile ducts and colonize the gallbladder or hepatic parenchyma and induce the formation of liver abscesses [6].

Throughout the migration of adult *A. lumbricoides* into the bile duct, the excretions of these parasites induce irritation, resulting in biliary colic, spasms of the sphincter of Oddi, and partial or complete biliary obstruction. In cases of co-infection, this progression may lead to such complications as cholangitis, cholecystitis, and inflammation of the pancreas [12,13,14]. Biliary ascariasis can manifest as abdominal pain, located in the right upper quadrant and lasting for several days, nausea and/or vomiting, general malaise, jaundice, and hepatomegaly [2,6]. Conjugated hyperbilirubinemia with elevated liver enzymes (ALT, AST, GGT, and ALP) in laboratory tests indicates the obstructive cause of jaundice. However, a significant elevation in ALP does not occur in all patients [9,15]. Our patient experienced recurrent episodes of abdominal pain in the right upper quadrant, vomiting, and even jaundice during the last episode. Conjugated hyperbilirubinemia and increased liver enzymes (ALT, AST, GGT) were observed in the patient’s laboratory tests. However, ALP levels remained constant, without exhibiting significant changes.

Once the worm enters the bile ducts, it can then exit the ducts within several days. We believe that while our patient was experiencing the first episode of the disease, *A. lumbricoides* entered the bile duct and spontaneously disappeared on their own without causing long-term consequences since previous symptoms did not reoccur about 6 months after the first painful episode. Researchers suggest that if a helminth within the bile ducts does not change its position within 10 days, it is then most likely to appear dead or macerated [7]. Within the bile ducts, *A. lumbricoides* may be present either alive or in various forms of decomposition, such as fragmented segments or remnants of tissue [16]. Upon the death of the parasites, mucosal inflammation, profuse exudation, and eosinophilic infiltration occur and cause a fibrotic response and subsequent bile duct stenosis [12]. Subsequent to the death of the worms within bile and liver ducts, sludge formation in the gallbladder occurs, potentially progressing to the development of gallstones over time [5].

We observed eosinophilia and a high concentration of IgE in the peripheral blood of our patient. Eosinophilia is often observed in the complete blood count, which usually reaches levels of 5–12 percent and sometimes as high as 30–50 percent in cases of bile duct ascariasis [14]. Eosinophilia usually occurs once the larvae migrate through the lungs, and it can also persist when adult worms relocate to the intestines, or when complications develop.

The diagnosis of ascariasis is confirmed after the detection of *A. lumbricoides* eggs in the stool. However, stool tests may often be negative [14]. In our case, the stool test was positive, together with *Ascaris lumbricoides* IgG4 antibodies being detected in the blood of our patient. The literature suggests that the anti-*Ascaris* IgG4 antibody is a very sensitive and specific marker for the diagnosis of *Ascaris* infection and is highly useful for conducting ascariasis screening [17].

The migration of parasites into the bile ducts is typically determined by imaging tests, such as ultrasound, magnetic resonance cholangiopancreatography, and endoscopic retrograde cholangiopancreatography.

Ultrasound is a safe, non-invasive, accurate, and accessible tool for the diagnosis of gallbladder and bile duct ascariasis. Sonography is a highly sensitive and specific diagnostic method (40–70 and 90 percent, respectively) [7,12], which enables the visualization of the parasite within the biliary system, as well as the tracking of its movement into and out of the ducts over time. A circular and elongated filling defect of the CBD is often observed during sonography. Although ultrasonography is assumed to be sensitive and specific, the quality of the examination also depends on the experience of the examining physician. When there is air present in the bile ducts due to prior interventions or because of the process of worm maceration, particularly when the worm is deceased, it may go unnoticed during sonography [7,12].

MRCP and ERCP should be performed to confirm the diagnosis if ultrasound examination is insufficient to prove the presence of the parasites. These tests are fundamentally different; however, their sensitivity and specificity are almost 100 percent for determining the cause of bile duct obstruction. Magnetic resonance cholangiography is an excellent option for diagnosing this parasitosis in the biliary tract (sensitivity and specificity of 100 percent) [7,12,18,19]. Endoscopic retrograde cholangiopancreatography serves a dual purpose, facilitating both diagnostic and therapeutic interventions. This method achieves optimal results in visualizing and extracting worms from bile ducts (with a sensitivity of 100 percent and specificity ranging from 94 to 95 percent) [5,7,12,19,20]. ERCP diagnostic accuracy is enhanced through direct visualization while serving as a curative procedure by facilitating the extraction of the identified parasite. All of the previously mentioned imaging methods were used to confirm the diagnosis of our patient. MRCP and ERCP demonstrated filling defects in the gallbladder and bile ducts, while ultrasonography did not identify any of these filling defects.

The treatment of biliary ascariasis includes conservative, endoscopic, and surgical methods [21]. Research demonstrates that the majority (90 percent) of children with biliary ascariasis respond to antihelminthic treatment within 3–5 days, but some patients do require surgical intervention [6]. During ongoing investigations, patients are advised to undergo conservative treatment, consisting of intravenous fluids, antispasmodics, and a course of antihelminthic drugs [9]. Pyrantel pamoate, mebendazole, albendazole, and levamisole are effective antihelminthic drugs [7,21,22]. If the patient’s clinical condition does not improve, or if complications arise as in the case of biliary ascariasis, it is advisable to discontinue conservative treatment and promptly initiate surgical intervention to mitigate potential complications [7,23]. Following the diagnosis of bile duct ascariasis, the optimal treatment approach involves the extraction of the parasite or of its remains from the bile ducts either via ERCP, or laparoscopic or open bile duct surgeries [2,5,21]. However, the surgical method is an alternative form of treatment when endoscopic deworming is not possible. In the case of our patient, ERCP successfully eliminated the remnants of a deceased *A. lumbricoides* individual, as opposed to an intact parasite. We consider that the removal of only parts of the parasite, as opposed to an intact individual, was supported by the patient receiving antihelminthic treatment prior to the procedure.

## 4. Conclusions 

It is essential to consider ascariasis as a differential diagnosis in children who complain of upper abdominal pain and develop jaundice not only in endemic regions, but also in areas with low prevalence of ascariasis. Clinical symptoms appear acutely and rapidly regress due to the active migration of *Ascaris* into and out of the bile ducts. Consequently, the disease may remain undiagnosed for an extended period of time. The diagnosis of biliary ascariasis requires common laboratory tests (complete blood count, liver enzymes, stool test), ultrasound, and—if further testing is required—MRCP. 

## Figures and Tables

**Table 1:** Results of laboratory tests performed on the patient.

Laboratory Tests	Reference Range	Results of the Laboratory Test			
		2nd Episode		3rd Episode	
		Day 1	Day 7	Day 1	Day 7
Complete blood count					
Erythrocytes (×10^12^/L)	4.2–5.3	4.50	n/a	4.52	n/a
Hemoglobin (g/L)	120–145	121	n/a	121	n/a
Leukocytes (×10^9^/L)	3.4–10.8	6.8	n/a	6.7	n/a
Neutrophils (%)	30–71	43.4	n/a	45.3	n/a
Lymphocytes (%)	17–58	41.5	n/a	40.0	n/a
Eosinophils (%)	0–4	5.3	n/a	8.5	n/a
Platelet count (×10^9^/L)	200–539	230	n/a	286	n/a
ESR (mm/h)	0–11	6	n/a	n/a	n/a
CRP (mg/L)	0–5	5	n/a	8	5
Liver function test					
Total bilirubin (µmol/L)	1.7–15.4	19.69	n/a	66.50	10.65
Conjugated bilirubin (µmol/L)	0–3.4	6.28	n/a	44.28	3.91
ALT (IU/L)	7–45	334	77	366	74
AST (IU/L)	8–50	378	30	256	29
ALP (IU/L)	69–325	323	334	332	n/a
GGT(IU/L)	3–22	176	93	216	123
SPA (%)	70–130	69	n/a	n/a	n/a
Alpha amylase (U/L)	21–110	171	91	306	109
IgE concentration (kU/L)	0–295	1192.9	n/a	n/a	n/a

ESR—erythrocyte sedimentation rate; CRP—C-reactive protein; ALT—alanine transaminase; AST—aspartate transaminase; ALP—alkaline phosphatase; GGT—gamma-glutamyltransferase; SPA—Owren’s Stago Prothrombin Assay, IgE—immunoglobulin E, n/a—not available.

## Data Availability

The original contributions presented in the study are included in the article, further inquiries can be directed to the corresponding author.
